# Is Omega-3 Fatty Acids Enriched Nutrition Support Safe for Critical Ill Patients? A Systematic Review and Meta-Analysis

**DOI:** 10.3390/nu6062148

**Published:** 2014-05-30

**Authors:** Wei Chen, Hua Jiang, Zhi-Yuan Zhou, Ye-Xuan Tao, Bin Cai, Jie Liu, Hao Yang, Charles Damien Lu, Jun Zeng

**Affiliations:** 1 Department of Parenteral and Enteral Nutrition, Peking Union Medical College Hospital, Beijing 100730, China; E-Mail: txchenwei@sina.com; 2 Department of Computational Mathematics and Bio-statistics, Metabolomics and Multidisciplinary Laboratory for Trauma Research, Sichuan Provincial People’s Hospital, Sichuan Academy of Medical Sciences, Chengdu 610101, China; E-Mails: cdzhouzhiyuan@gmail.com (Z.-Y.Z.); taoyx@163.com (Y.-X.T.); bin.cai@traumabank.org (B.C.); liuxiaoxiao1s@126.com (J.L.); hao.yang@traumbank.org (H.Y.); damienla@gmail.com (C.D.L.); zengjun@medmail.com.cn (J.Z.); 3 Department of Trauma Surgery, Sichuan Provincial People’s Hospital, Sichuan Academy of Medical Sciences, Chengdu 610101, China; 4 Department of Clinical Nutrition, Xin Hua Hospital, School of Medicine, Shanghai Jiaotong University, Shanghai 200092, China; 5 Department of Biochemistry, Luzhou Medical College, Luzhou 646000, China

**Keywords:** omega-3 fatty acids, severe illness, parenteral nutrition, enteral nutrition, meta-analysis

## Abstract

Objective: To systematically review the effects of omega-3 poly unsaturated fatty acids (FA) enriched nutrition support on the mortality of critically illness patients. Methods: Databases of Medline, ISI, Cochrane Library, and Chinese Biomedicine Database were searched and randomized controlled trials (RCTs) were identified. We enrolled RCTs that compared fish oil enriched nutrition support and standard nutrition support. Major outcome is mortality. Methodological quality assessment was conducted based on Modified Jadad’s score scale. For control heterogeneity, we developed a method that integrated I^2^ test, nutritional support route subgroup analysis and clinical condition of severity. RevMan 5.0 software (The Nordic Cochrane Centre, Copenhagen, Denmark) was used for meta-analysis. Results: Twelve trials involving 1208 patients that met all the inclusion criteria. Heterogeneity existed between the trials. A random model was used, there was no significant effect on mortality RR, 0.82, 95% confidence interval (CI) (0.62, 1.09), *p =* 0.18. Knowing that the route of fish oil administration may affect heterogeneity, we categorized the trials into two sub-groups: parenteral administration (PN) of omega-3 and enteral administration (EN) of omega-3. Six trials administered omega-3 FA through PN. Pooled results indicated that omega-3 FA had no significant effect on mortality, RR 0.76, 95% CI (0.52, 1.10), *p =* 0.15. Six trials used omega-3 fatty acids enriched EN. After excluded one trial that was identified as source of heterogeneity, pooled data indicated omega-3 FA enriched EN significant reduce mortality, RR=0.69, 95% CI [0.53, 0.91] (*p =* 0.007). Conclusion: Omega-3 FA enriched nutrition support is safe. Due to the limited sample size of the included trials, further large-scale RCTs are needed.

## 1. Introduction

Omega-3 fatty acid is a group of essential fatty acids, which constitutes cellular membrane and possesses merits on membrane stabilization and immune regulation [1,2]. Since the 1980s, there has been abundant research using omega-3 FA to prevent cardiovascular disease (CVDs) and related comorbidities [3,4,5]. In the past decade, more evidence has emerged on role of omega-3 fatty acid enriched nutrition supplements for the treatment of various diseases [6,7,8]. 

In an earlier study we found that omega-3 fatty acids (FA) enriched parenteral nutrition could improve outcome of selective surgical patients [9]. Recently published clinical trials are raising controversy on the application of omega-3 fatty acids as pharmaceutical nutrients in critically ill patients [10,11,12]. Although some of these studies found that omega-3 FA plays positive role on immune-regulation, organ protection and metabolism, the others found that it might not improve clinical outcomes. In 2011, a large-scale, prospective randomized control trial (RCT) found that the administration omega-3 FA through enteral nutrition is associated with an increase of death risk [13]. In short, the definitive conclusion on the efficacy of using omega-3 FA for critically ill patients still does not exist. 

In terms of the dilemma on omega-3 FA for critical illness, a single trial may be limited from its design, sample size, bias, and patient type. To answer the question “Is omega-3 FA safe for critically ill patients”, a systematic review is urgently needed which is what we present here. 

## 2. Material and Method

### 2.1. Literature Search Strategy

We retrieved relevant articles published before 31 December, 2013, from MEDLINE (through PubMed), SCI (through ISI), Cochran Library and Chinese Biomedicine Database using the following keywords: ((omega-3 fat emulsion or fish oil emulsion) or (*n*-3 fatty acids and parenteral)) and (critical illness or intensive care unit or severe illness). Detailed search strategy for each database is listed in Table 1. In addition, we searched the following journals manually: Journal of American Clinical Nutrition (January 1990 to December 2013), Journal of Parenteral and Enteral Nutrition (January 1990 to December 2013), Nutrition (January 1990 to December 2013), Clinical Nutrition (January 1990 to December 2013), and Chinese Journal of Clinical Nutrition (1993 to December 2013). We also contacted enteral or parenteral omega-3 product producers and study investigators of published randomized trials. Whenever necessary, we consulted other relevant principal investigators and experts.

**Table 1:** Literature research strategy, databases and key words.

Database	Search Strategy
MEDLINE (Through PubMed)	(“Omega-3 fatty acids” [Title/Abstract] OR “fish oil” [Title/Abstract] OR “*n*-3 fatty acids” [Title/Abstract]” OR “eicosapentaenoic acid” [Title/Abstract] OR “docosahexaenoic acid” [Title/Abstract]) AND (“parenteral nutrition” [Title/Abstract] OR “TPN” [Title/Abstract] OR “PN” [Title/Abstract] OR “enteral nutrition” [Title/Abstract] OR “EN” [Title/Abstract]) AND (“critical” [Title/Abstract] OR “severe” [Title/Abstract] OR “intensive care” [Title/Abstract] OR “ICU”)
Cochrane Library	(“Omega-3 fatty acids” OR “fish oil” OR “*n*-3 fatty acids”) AND (“parenteral nutrition” OR “TPN” OR “PN” OR “enteral nutrition” OR “EN”) AND (“critical” OR “severe” OR “intensive care” OR “ICU”)
Chinese Biomedicine Database and CNKI	(“Yu You” OR “Omega-3” OR “Duo Bu Bao He Zhi Fang Suan”) AND (“Chang Wai Ying Yang” OR “TPN” OR “PN” OR “Chang Nei Ying Yang” OR “EN”) AND (“Wei Zhong Bing” OR “Wei Zhong Zheng” OR “ICU”)

### 2.2. Study Selection

#### Inclusion and Exclusion Criteria

Inclusion and exclusion criteria were discussed to reach consensus within the reviewer team. Two investigators (Wei Chen. and Hua Jiang.) independently selected trials and extracted data on a non-blinded basis. Differences in interpretations were resolved through group discussions.

Inclusion criteria: (1) Study design: Only RCTs with parallel control groups were selected, excluding self-control or crossover trials; (2) Type of patients: adult (age ≥ 18 year) critically ill patients who were admitted to intensive care units or who had APACHE III scores 10 or higher; (3) Intervention: omega-3 fatty acids enriched nutrition regimen was the only difference between experimental and control groups; (4) studies that administered omega-3 fatty acids through enteral or parenteral routes were included; (5) Outcome: mortality.

Exclusion criteria: (1) non-randomized design or pseudo-randomization; (2) studies that did not address any primary or secondary outcome as mentioned above; (3) crossover studies; (4) studies on healthy volunteers; (5) for Chinese studies, where there is a high prevalence of quasi-randomization [14], and additional step was taken. For those studies that did not describe the randomization method or where we questioned the soundness of the randomization method, we attempted to contact the original authors. If the original authors did not provide a response or the randomization method proved inadequate, the articles were excluded.

Omega-3 fatty acids could be administered through parenteral or enteral route, thus, a challenge for the researchers of systematic review is how to control the internal heterogeneity in study design. Considering omega-3 is the key component of nutrition intervention of the study, we decided that either route is acceptable. To minimize the impact from the difference in energy intakes, we developed a method to weigh the variants between the trials, which are discussed in detail in the “Measurements and Analytical Methods” section. 

### 2.3. Methodological Quality Evaluation

The methodological quality assessment table below is based on the Cochrane Reviewers’ Handbook [14] and a modified Jadad scale (Table 2) [15,16]. The original Jadad scale is a 5-point system. Because inadequate concealment of treatment allocation has been associated with an exaggeration of treatment effects, we decided to adopt a modified Jadad score scale, which assigns a maximum of 2 points for concealment [16,17]. Under this system, a maximum score of 7 was assigned. Studies with a score of ≥4 were considered to be high-quality studies.

**Table 2:** Modified Jadad Scores Scale.

	Items	Points
Randomization	Appropriate	2
	Did not describe the details of randomization	1
	Inappropriate	0
Concealment	Appropriate	2
	Did not describe the details of concealment	1
	Inappropriate or no concealment	0
Blinded	Appropriate	2
	Did not describe the details of blinded	1
	Inappropriate or not blinded	0
Withdraw or drop-out	Described	1
	Did not describe	0

### 2.4. Measurements and Analytical Methods

We assessed heterogeneity in two steps. First we used an I^2^ measure, with I^2^ > 75% indicating high heterogeneity, I^2^ between 75% and 50% indicating moderate heterogeneity, I^2^ between 49% and25% indicating low heterogeneity [18]. We combined data from all the trials with minimal, low or no heterogeneity using the Mantel-Haenszel fixed effect model [18]. In addition, as confirmation, we also used the random effect model when I^2^ > 0. For those with moderate or high heterogeneity, we combined data using random effects models. Whenever heterogeneity was found, the sources were analyzed, and stratified analysis was conducted to exclude the highly variable trial(s) and to decrease heterogeneity (e.g., relatively severe patient conditions *vs*. relatively moderate patient conditions, relatively small sample sizes *vs*. large sample sizes, or high methodological quality *vs*. low methodological quality). When the I^2^ value was substantially lower in each stratified analysis, we compared the result of stratified analysis with the result of overall analysis. In such circumstances, our conclusion would be consistent with the result based on the homogenous and more methodologically rigorous trials [16]. Secondly, considering mortality is highly depends on the severity of condition when the patients were admitted and it is a core issue for any critical illness study, we therefore introduced a quantitative severity variant measurement method. For any trial that was demonstrated by I^2^ test as a source of heterogeneity, we calculated expected mortality by means of the adjustment linear regression model [19,20,21]. The formula of Observed/Expected Ratio (O/E Ratio) calculation is:

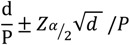
(1)
where, *d* is amount of death, *P* is sample amount and *Z*_α/2_ is α/2 median of normal distribution.

Whenever the actual mortality of such a trial was significantly higher or lower than expected mortality, we concluded the trial was substantially heterogeneous and should be excluded from data synthesis of meta-analysis. 

For mortality, a risk ratio (RR) with a 95% confidence interval (CI) was calculated. RR and its 95% CI < 1 indicate significantly lower incidences in the group with omega-3 fatty acids administration and was designated as *p* < 0.05; whereas a RR and its 95% CI > 1 favor the control group and was designated *p* < 0.05. When there was no statistical significance when a RR of 1 or its 95% CI crossed 1 between groups, it was designated as *p* > 0.05.

All statistical processes were conducted using a high-performance-computing platform at the Metabolomics and Multidisciplinary Laboratory of Sichuan Academy of Medical Sciences. Review Manager 5.1 statistical software (Cochrane Collaboration) was used for the meta-analysis [22]. For linear regression modeling, we used R (Version 2.15.2).

We followed the PRISMA (Preferred Reporting Items for Systematic Reviews and Meta-Analyses) Statement to report the research protocol, outcome and relevant items in this systematic review [23].

## 3. Results

### 3.1. Study Identification and Selection

**Figure 1:**
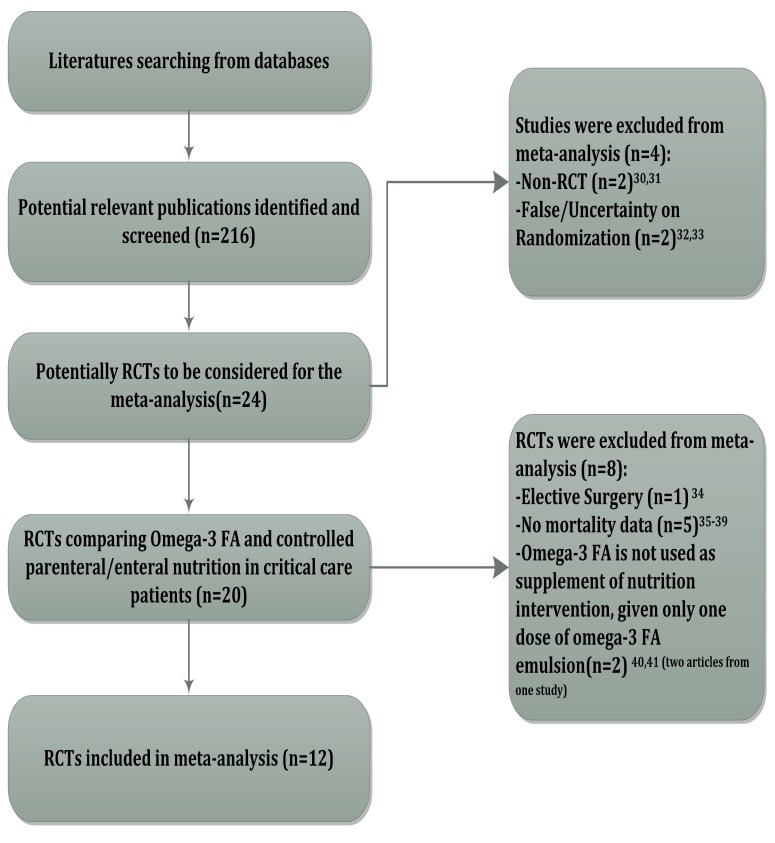
Literature searching and selection.

In total 216 potentially relevant titles, abstracts, and articles were found. Initial screening resulted in 23 candidate studies (24 articles, with two belonging to one study) [11,12,13,24,25,26,27,28,29,30,31,32,33,34,35,36,37,38,39,40,41,42,43,44]. Figure 1 shows the details of the selection process and the reasons for exclusion. After further screening, there were twelve trials (including one Chinese study) that met all inclusion criteria and were included in the final systematic review [11,12,13,24,25,26,27,28,29,30,31,32]. The characteristics of these included trials are listed in Table 3.

**Table 3:** RCTs of Omega-3 fatty acids supplementation for severe patients.

Author, year	M.J.S	P.T.	N.O.P(ITT)	R.O.F	R.O.A.	Mortality *n*/*N* (ITT)		Calorie administered (kcal/day)	
						Omega-3 Group	Control Group	Omega-3 Group	Control Group
Gadek, J., 1999 [24]	5	ARDS	146	EN	EN	11/70	19/76	1728 ± 74	1692 ± 60
Grecu, I., 2003 [25]	5	Abdominal Sepsis	54	PN	PN	2/28	2/26	NR	NR
Mayer, K., 2003 [26]	4	sepsis	21	PN	PN	5/10	3/11	NR	NR
Pontes-Arruda, A., 2006 [27]	5	sepsis	103	EN	EN	18/55	25/48	1621 ± 48	1647 ± 74
Singer, P., 2006 [28]	5	ALI	100	EN	EN	13/46	28/49	1624 ± 512	1420 ± 437
Friesecke, S., 2008 [12]	7	Patients in medical ICU	166	PN + EN	PN	18/83	22/82	22.2 ± 5.5 (kcal/kg/day)	21.6 ± 5.6 (kcal/kg/day)
Wang, X., 2008 [11]	4	SAP	56	PN	PN	0/28	2/28	27(kcal/kg/day)	27 (kcal/kg/day)
Barbosa, V., 2010 [29]	3	sepsis	23	PN	PN	2/13(5D), 4/13(28D)	1/10(5D), 4/10(28D)	2057 ± 418	1857 ± 255
Grau-Carmona, T., 2011 [30]	5	sepsis	132	EN	EN	11/61	11/71	1718 (1189–1956)	1599 (1351–1976)
Gupta, A., 2011 [31]	7	ARDS	61	EN	PN	7/31	13/30	1800	1800
Stapleton, R.D., 2011 [32]	5	ALI	90	EN	EN	9/41(14 days) 9/40(60 days)	10/49(14 days) 11/45 (60 days)	NR	NR
Rice, T., 2011 [13]	7	ALI	272	EN	EN	38/143	21/129	800–900	800–900

Abbreviations: ALI: acute lung injury; ARDS: acute respiratory dysfunction syndrome; EN: enteral nutrition; PN: parenteral nutrition; ITT: intention to treat; SAP: severe acute pancreatitis; NR: not reported; M.J.S: Modified Jadad Score; P.T.: Patient Type; N.O.P: No. of patients; ITT: Intention to treat; R.O.F: Route of Feeding; R.O.A.: Route of Omega-3 administered.

### 3.2. Primary Outcome: Mortality

#### 3.2.1 Overall Analysis

**Figure 2:**
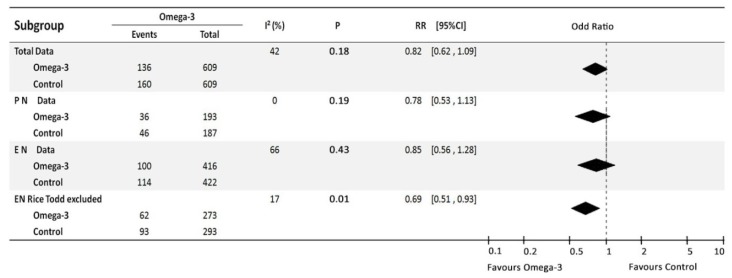
Effect of Omega-3 FA administration on mortality of severe patients.

The twelve RCTs presented mortality data involving a total of 1218 patients. Analysis found that heterogeneity existed between trials (I^2^ = 42%, *p =* 0.06). A random model was used to aggregate the data, and there was no significant effect on mortality: RR, 0.82, 95% CI (0.62, 1.09), *p =* 0.18 (Figure 2).

Considering the route by which Omega-3 FA is administered may be a source of heterogeneity, we categorized the trials into two sub-groups: parenteral fish oil administration and enteral fish oil administration.

#### 3.2.2. Sensitivity Analysis for All-Included Trials

**Figure 3:**
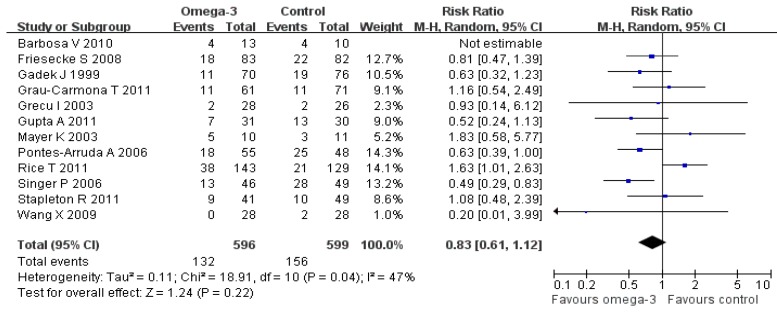
Sensitivity analysis: high quality trials.

As methodological quality may also be a source of heterogeneity, trials with quality score (modified Jadad Scale) of ˂ 4 were excluded and the remaining trials were then combined. The study by Barbosa *et al.* [29] is a relatively low in methodological quality and was excluded, and the remaining eleven trials were included. The result of the heterogeneity test indicated low heterogeneity (*p =* 0.04, I^2^ = 47%). A random effect model also revealed that there was no statistically significant effect on mortality (RR, 0.83, 95% CI 0.61–1.12, *p =* 0.22; I^2^ = 47%; Figure 3). This result is consistent with the overall analysis.

#### 3.2.3. Sub-Group Analysis

##### 3.2.3.1. Parenteral Omega-3 Studies

Six trials administered omega-3 fatty acids through the parenteral route, and no heterogeneity was found (I^2^ = 0, *p =* 0.54). We used fixed model to aggregate data. Results indicated that omega-3 FA had no significant effect on mortality: RR 0.76, 95% CI (0.52, 1.10), *p =* 0.15.

##### 3.2.3.2. Sensitivity Analysis for PN Omega-3 Studies

One low methodological study: Barbosa study was excluded and aggregated remaining trials. Five trials were included and no heterogeneity was found (I^2^ = 0, *p =* 0.45). We used fixed model to aggregate data. Results indicated that omega-3 FA had no significant effect on mortality: RR 0.86, 95% CI (0.54, 1.37), *p =* 0.54. This result is consistent with overall analysis of the parenteral omega-3 subgroup.

##### 3.2.3.3. Enteral Omega-3 Studies

Six trials used omega-3 fatty acids enriched enteral nutrition. I^2^ test found significantly high heterogeneity (I^2^ = 66%, *p =* 0.01). Further analysis found that the Rice T. *et al*. study might be the source of heterogeneity. There are two major important differences between the Rice T. study and the rest of the trials: (1) the patient type; and (2) significantly lower calorie intake. The patients enrolled in the Rice T. study were much more severely ill (mean APACHE III scores: 93.8 in omega-3 group *vs*. 91.8 in control group) than those in the other enteral omega-3 studies. 

### 3.3. An Advanced Heterogeneity Test: O/E Ratio

**Figure 4:**
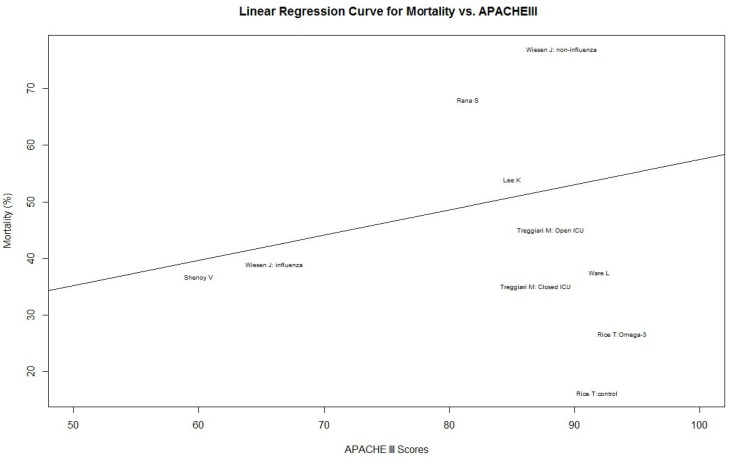
Heterogeneity Analysis: Linear Regression Curve for Impact on Mortality and APACHE III Scores: The horizontal axis shows the APACHE III scores, and the vertical axis shows the mortality (%). According to the data of ALI studies, a linear regression model is set up based on the APACHE III scores and the mortality. The regression equation is expressed by the straight line in the figure.

**Figure 5:**
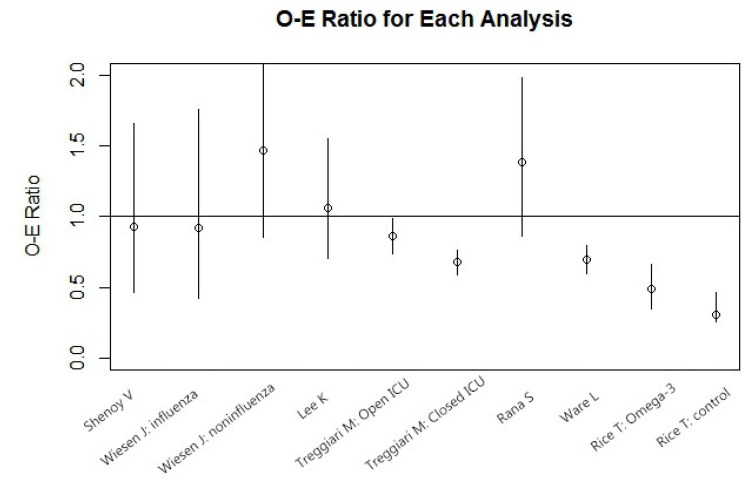
Heterogeneity Analysis: Observed/Expected Ratio (O/E Ratio) of Acute Lung Injury Studies: The vertical axis shows the O/E Ratio. The O/E Ratio of both Omega-3 group (0.48, 95% CI: 0.34–0.67) and control group (0.30, 95% CI: 0.19–0.46) in Rice T. *et al*. [13] study are far below 1, which indicate that the patients in the Rice T. *et al*. study are in significantly more severe condition, but have significantly lower mortality.

In order to check for heterogeneity in the mortality of the studies, we employed the method of O/E Ratio to obtain the O/E Ratio and 95% CI. We made a further systematic literature search and aimed to identify relation between APACHE III scores and mortality of acute lung injury (ALI). We found six ALI studies (RCT or Prospective Cohort) that reported APACHEIII scores (Table 4) [45,46,47,48,49,50]. Result in this literature search found that the patients in the Rice, T.’s study [13] were the most severely ill (mean APACHE III scores: 93.8 in omega-3 group *vs*. 91.8 in control group) but had the lowest mortality (mortality in other ALI trials: 35%–77%; mortality in Rice, T. *et al*. [13]: 16.3% for control group and 26.6% for omega-3 EN fed group) (Figure 4). We established a linear regression model to calculate expected mortality in APACHE III scores categorized and introduced O/E ratio as a further quantitative heterogeneity evaluation tool. We found that in an O/E ratio test, the Rice T. study was significantly different from all other ALI study (Figure 5). This means that the Rice, T.’s study is not only different from the other omega-3 studies we enrolled in this systematic review, but also significantly different from most recently ALI studies. In summary, the patients in the Rice, T. *et al*. study, although categorized as “ALI”, were substantially different from those in the others trials [13]. In addition, compared with other enteral omega-3 trials, the Rice, T. *et al*. study fed patients significantly lower energy (accumulated calorie intake of first 7 days is: 5920 ± 3624 kcal (omega-3 FA group) *vs*. 6072 ± 3602 kcal (control group) translating into an average energy intake around 800–900 kcal/day in the first 7 days of the study) [13,51].

**Table 4:** Mortality of acute lung injury (ALI) studies using APACHE III as severity condition measures.

ALT Study	APACHE III	Sample size	Mortality (%)
Shenoy, V. [45]	60	30	36.7
Wiesen, J.: Influenza [46]	66	23	39.0
Wiesen, J.: Non-influenza [46]	89	22	77.0
Lee, K.[47]	85	50	54.0
Treggiari, M.: Open ICU [48]	88.1	391	45.0
Treggiari, M.: Closed ICU [48]	86.9	684	35.0
Rana, S.[49]	81.5	38	68.0
Ware, L.[50]	92	528	37.5
Rice, T.: Omega-3[13]	93.8	143	26.6
Rice, T.: Control [13]	91.8	129	16.3

We therefore decided to exclude Rice, T. *et al*. [13] study from EN-fed omega-3 meta-analysis. When this study was excluded and aggregated the remaining enteral omega-3 FA studies, we found there was no significant heterogeneity between these five trials (I^2^ = 17%, *p =* 0.31). The fixed model was used to pool the data involving a total of 566 patients. The result indicated that omega-3 fatty acids enriched enteral nutrition could significant reduce mortality, RR = 0.69, 95% CI (0.53, 0.91) (*p =* 0.007) (Figure 3). To Confirm, we also employed the random model and found similar result: RR = 0.69, 95% CI (0.51, 0.93) (*p =* 0.01).

## 4. Discussion

Big controversy rose on the issue of omega-3 fatty acids enriched enteral nutrition when a large-scale, double-blinded controlled study by Rice T. *et al*. was published in 2011 [13]. It concluded that using omega-3 fatty acids enriched enteral nutrition regimen for acute lung injury patients would increase mortality risk. Because omega-3 fatty acids had been seen as an important achievement of pharmaceutical nutrient in the last decade, this result shocked the field of clinical nutrition. For clinical practitioners, a systematic evaluation based on best evidence was urgently needed. This was the reason we undertook this systematic review and meta-analysis.

We included twelve RCTs in our systematic review. Based on the result of meta-analysis we found that an omega-3 enriched nutrition regimen would not increase death risk in critically ill patients, whether administered by enteral or parenteral route. However, the result of the meta-analysis also found that omega-3 enriched nutrition regimen does not provide protective effect to patients in their clinical outcome.

Although in total the pooled results were not confirmatory, we had several interesting findings when we looked closely at the trials. One of very important findings was that the impact of omega-3 fatty acids enriched EN and mortality were related to calorie intake. The major controversy on omega-3 fatty acids in critically ill patients arose after Rice T. *et al*. reported patients who were administered EN regimen enriched by omega-3 fatty acids, gamma-linoleic acid and antioxidant had higher death risk [13]. They drew the conclusion that the harmful effect mainly came from omega-3. We compared the Rice T. *et al*. study and others and found something different: calorie was the culprit. Patients were given hypocaloric EN in the Rice T. *et al*. study [13]. The calorie intake in the first 7 days were quite low, average calorie intake was 800–900 kcal/day in first 7 days of the study. In contrast, in the remaining five enteral omega-3 FA trials patients were provided with significantly higher calories. When we pooled these five trials, we found patients who were in omega-3 intervention regiment had lower mortality risk (RR = 0.44, 95% CI [0.45, 0.82] (*p =* 0.001), Figure 3). It is well documented that deficit of calorie is associated with increased ICU mortality and morbidity [52,53,54,55]. Hence, we have reasonable doubt that the increasing of mortality in Rice T. study is more related with hypocaloric feeding other than omega-3 fatty acids administration.

We were interested in the Rice T. *et al*. study and provided a *post hoc* analysis for the heterogeneity between it and other enteral fish oil studies [13]. Through our linear regression model, we found that the mortality of the Rice T. *et al*. study was significantly lower than the other enteral fish oil studies, and it also posed the lowest mortality in all major acute lung injury studies [13]. In another word, the Rice T. *et al*. study was not only different from other enteral fish oil studies; it was also quite different from other acute lung injury studies. 

In summary, considering the above, and that the larger sample size of the other full calorie intakes of enteral fish oil studies (566 *vs*. 272 of Rice T. *et al*. [13]), together with the documented strong harmful effect of calorie deficiency in ICU, we concluded that enteral enriched omega-3 fatty acids may safe when calorie supplementation is adequate. But this conclusion urgently needs to be verified by future study.

The main limitation of our study is the limited sample size of the included trials. Most of the trials that included in systematic review are enrolled no more than 100 patients (median sample size is around 21–65). Even when we pooled all trials together, there were only 1218 patients, which was not enough for demonstrate mortality risk (estimated power 0.80, need no less than 2156, and current sample size only reached 60% of this scale). Further large scale randomized clinical trials are urgent needed.

Heterogeneity of patient types, dose and the route of omega-3 FA intervention administration was another limitation. Our study found clues indicating that the effects of omega-3 may be related to the energy supplementation. It reflected how complicated the omega-3 FA’s effects are on adjusting the pathophysiological conditions for critically ill patients. Unfortunately, this not enough attention has been given to this complicated scenario in published studies. For future researchers, a better design should carefully define these aspects. 

## 5. Conclusions

In conclusion, we found omega-3 FA enhanced nutrition support may safe for critically ill patients if the energy provision is sufficient. Compared with providing omega-3 FA via parenteral route, omega-3 enriched enteral nutrition may be favorable with decreased death risk. However, this is not confirmatory as the sample size, dosage of omega-3 FA, and accompanied energy provision are different in current RCTs. 
